# Changing peer review practices: transforming roles and future challenges

**DOI:** 10.2340/17453674.2025.44353

**Published:** 2025-07-18

**Authors:** Serge P J M HORBACH, Søren OVERGAARD

**Affiliations:** 1 Institute for Science in Society, Radboud University, Netherlands; 2 Copenhagen University Hospital Bispebjerg, Department of Orthopaedic Surgery and Traumatology, University of Copenhagen, Denmark

## The peer review process in the past and present

Despite many critiques of the system being slow, biased, expensive, and not always effective in distinguishing good from problematic research [1-3], peer review is still considered the hallmark of scholarly communication practices. By many, it is even considered a defining element of science.

While it tends to be referred to as a single procedure, the peer review system has historically been characterized by a wide diversity of practices and procedures [4,5]. Starting off from a procedure to select manuscripts worthy of publication within the limited number of pages of journals, usually performed by an individual editor in chief or small editorial board, the system began introducing external reviewers as science diversified and specialized [6]. *Acta Orthopaedica* started with external reviewers in 1971. Specialized reviewers were required for their expertise to assess increasingly niche topics, which could no longer be done by a small group of editors.

With the introduction of external reviewers [7], various concerns that needed to be tackled emerged. This included concerns over fairness and bias, diversity, and proper recognition [8-10]. Such concerns led to a diversification of review procedures, with journals either using single, double, or even triple anonymous review procedures [11], and recently also using open review procedures in which identities of those involved or the content of the review reports are openly disclosed [12]. *Acta Orthopaedica* also engages in these practices, disclosing reviewer identities in published articles, unless reviewers actively opt out. In addition, procedures to tackle conflicts of interest, attempts to provide recognition and reward for reviewers, or formats using diverse review criteria have led to further experimentation with review and editorial procedures [13].

Ultimately, these all relate to the questions of who is a qualified reviewer, what should be reviewed, and how can this best be done. Such concerns remain very timely and relevant today, for example when increased levels of interdisciplinarity and single manuscripts crossing disciplinary boundaries raise the question of who can rightfully be considered a “peer,” sufficiently qualified to assess a manuscript.

## Changing editorial practices

So, even if editorial practices have never been homogeneous, scholarly peer review is currently witnessing another wave of many and rapid transformations, increasingly fueled by developments in AI and other technologies.

With the introduction of novel, mostly AI-based technologies, another wave of developments and variations may be possible in the future. These technologies have been implemented to support or conduct various “technical” checks including those for text and image duplication [14], inappropriate references, alignment with reporting standards, and errors in statistical analyses or reporting [15]. However, these tools are also increasingly being used for other tasks, including some that directly relate to the content or process of manuscript peer review. Think for example of tasks like selecting appropriate reviewers [16], writing review reports, or analyzing manuscripts to identify strengths and weaknesses [17].

These new technologies and tools have several obvious advantages, and they potentially address several long-standing challenges of the peer review system. This includes reducing the time required for initial checks, enhancing the detection of plagiarism or other research misbehaviors, and addressing the issue of reviewer fatigue [18,19]. In several ways, these tools have the potential to contribute to fighting research misconduct and boosting efficiency, leading some to argue that a fully automated future of editorial processes may be on the horizon [20]. However, the ability of large language models to write high-quality reviews is still hotly debated [17].

Apart from efficiency considerations, these technologies also have more fundamental implications. One of them comprises a restructuring of the roles and responsibilities of actors involved in the review and editorial process. Editors, who traditionally managed the entire review process, now share responsibilities with automated systems that can handle initial checks and reviewer selection. This shift allows editors to focus more on strategic decisions and the overall direction of the journal, but also brings risks in terms of quality and integrity [21].

Reviewers, on the other hand, may find their roles shifting towards more interpretative and evaluative tasks, as routine checks are automated. This could contribute to yet another wave of specialization of the review process, with reviewers contributing to the assessment of more specific aspects of manuscripts. Potentially, this can lead to a more efficient use of their expertise, and some claim it would lead to more objective review [22]. However, these tools also introduce new dynamics in defining what constitutes quality and integrity in research [16]. They implicitly set benchmarks for originality, relevance, and adherence to reporting standards, all informed by their training data and therefore necessarily reproducing past quality standards [19].

## Diverse implications for diverse journals

For several reasons, we might expect the emergence of these new technologies to play out differently for diverse journals.

First, we should note that the availability of these systems and tools is not equally distributed over academic outlets. As they tend to require substantial resources and investments, their availability remains largely restricted to large, typically commercial, publishers or other well-resourced journals. New AI tools, such as ChatGPT or other general-purpose tools, are sometimes freely available, but they come with the risk of breaching confidentiality as they use input for training data. Alternatively, in-house models can be created, or company protection services can be negotiated, but these require substantial resources that are only available to some publishers or journals.

With quality checks and efficient handling of papers being prime markers of journal “quality” [23], this skewed ability to integrate new tools in editorial processes runs the risk of strengthening the monopoly position of large publishers to the detriment of smaller, community-owned journals and publishers [24].

From the perspective of the *Acta Orthopaedica* editorial team, we want to be efficient, fair, and produce high-quality reviews. Only those manuscripts with a fair chance of being published should go into review. This requires a good screening process. The journal currently has the possibility to perform plagiarism checks on each manuscript and would be interested in tools for research integrity checks, such as checking for fabrication of data and potential involvement of paper mills. All the tools should protect the manuscript and safeguard confidentiality. If implemented well, these tools will make the editorial processes more homogeneous and efficient. Regarding the review process, better screening tools for use on reporting guidelines, registration of protocols, and statistical analysis plans would be helpful.

AI and other technologies will have their role in the future peer-review process, which has to be defined and developed, including how to implement it in medical writing [25]. However, the valuable work done by editors and reviewers will still be needed.

## Notes

Handling co-editor: Li Felländer-Tsai
